# Plasma-activated medium triggers cell death and the presentation of immune activating danger signals in melanoma and pancreatic cancer cells

**DOI:** 10.1038/s41598-019-40637-z

**Published:** 2019-03-11

**Authors:** Amalia Azzariti, Rosa Maria Iacobazzi, Roberta Di Fonte, Letizia Porcelli, Roberto Gristina, Pietro Favia, Francesco Fracassi, Ilaria Trizio, Nicola Silvestris, Gabriella Guida, Stefania Tommasi, Eloisa Sardella

**Affiliations:** 1 Experimental Pharmacology Laboratory, IRCCS Istituto Tumori Giovanni Paolo II, Viale O. Flacco, 65, 70124 Bari, Italy; 2 Institute of Nanotechnology, National Research Council of Italy (CNR-NANOTEC), c/o Department of Chemistry, University of Bari “Aldo Moro” via Orabona 4, Bari, 70126 Italy; 3 Department of Biosciences, Biotechnologies and Biopharmaceutics, University of Bari Aldo Moro via Orabona 4, Bari, 70126 Italy; 4 Department of Chemistry, University of Bari Aldo Moro Via Orabona 4, Bari, 70126 Italy; 5 Scientific Direction, IRCCS Istituto Tumori Giovanni Paolo II, Viale O. Flacco, 65, 70124 Bari, Italy; 6 Department of Basic Medical Sciences, Neurosciences and Sense Organs -University of Bari Aldo Moro via Orabona 4, Bari, 70126 Italy; 7 Molecular Diagnostics and Pharmacogenetics Unit, IRCCS Istituto Tumori Giovanni Paolo II, Viale O. Flacco, 65, 70124 Bari, Italy

## Abstract

Over the past decade, cold atmospheric plasmas have shown promising application in cancer therapy. The therapeutic use of plasma-activated media is a topic addressed in an emerging field known as plasma pharmacy. In oncology, plasma-activated media are  used to harness the therapeutic effects of oxidant species when they come in contact with cancer cells. Among several factors that contribute to the anticancer effect of plasma-activated liquid media (PALM), H_2_O_2_ and NO derivatives likely play a key role in the apoptotic pathway. Despite the significant amount of literature produced in recent years, a full understanding of the mechanisms by which PALM exert their activity against cancer cells is limited. In this paper, a sealed dielectric-barrier discharge was used to disentangle the effect of reactive nitrogen species (RNS) from that of reactive oxygen species (ROS) on cancer cells. Two cancers characterized by poor prognosis have been investigated: metastatic melanoma and pancreatic cancer. Both tumour models exposed to PALM rich in H_2_O_2_ showed a reduction in proliferation and an increase in calreticulin exposure and ATP release, suggesting the potential use of activated media as an inducer of immunogenic cell death via activation of the innate immune system.

## Introduction

In 1928, plasma was defined as the fourth state of matter: an ionized gas containing atoms, radicals, ions and molecules in ground and excited states, with an equal density of negative and positive particles^1^. Depending on how the energy is parted among the species of the system and among their degrees of freedom, plasma is usually classified as thermonuclear, thermal (equilibrium) or cold (non-equilibrium) plasmas^2,3^. Cold atmospheric plasma (CAP) indicates that the kinetic energy of the gas atoms, molecules, and ions is lower than that of the electrons, which results in a minimal temperature increase when compared to room temperature^4^.

Over the past decade, owing to advanced technology that allows plasma to be sustained at atmospheric pressure and room temperature, CAP has shown remarkably selective effects on biological systems both *in vitro* and *in vivo*. Direct (topical) plasma treatment of xenografted tumours as well as dermal treatment of wounds and other ailments in mammalian models have been extensively reported^5,6^. When in contact with living matter, plasma can promote inactivation of microorganisms, stimulation of cell proliferation and tissue regeneration and inactivation of cancer cells by initializing apoptosis^5^. Based on these results, 15 years ago, E. Stoffels, with her pioneering paper, created the basis for a new interdisciplinary field of research called plasma medicine: direct application of CAP, in both *in vitro* and *in vivo* models, to study its therapeutic potential^3,7,8^. CAP for plasma medicine is generally generated by two major types of devices: (1) plasma jets and (2) dielectric-barrier discharges (DBDs)^3,9^. In jets, the plasma is generated remotely, and the plasma products are delivered to the biological target via carrier gas. DBDs generate plasma either remotely, similar to the plasma jet, or directly at the surface to be treated, implying that living tissue or the cell layer itself is used as one of the electrodes directly participating in the plasma process.

Although evidence in the literature suggests that plasma-generated chemical reactive species are responsible for inducing certain cellular behaviours, plasma is composed of multiple effectors whose interaction with cells should not be overlooked. Examples of this are observed when the plasma-associated pulsed electric field promotes cell membrane electroporation, apoptosis and necrosis; UV light emitted from plasma induces DNA damage and cell death^10^. Regardless of the configuration used when plasma sources are interfaced with a biological system, the conductivity of the system can actively influence the process. During plasma processing, some current may flow through the living tissue electrode in the form of a small conduction current, displacement current or both^3^. Recent literature has shown that the role of each plasma effector can be studied and that reactive oxygen/nitrogen species (RONS), which are produced in the plasma phase, play a pivotal role in determining cellular effects^10^. A cascade of chemical reactions involving the active species generated in the plasma occurs at the liquid/gas interface, the macroscopic effect of which is to enrich the liquid of RONS. Active plasma species diffused in the liquid actually determine the nature and density of secondary species that, in turn, interact with cells and tissues^11^.

Starting from these indications, in the last three years, a new research field within plasma medicine has emerged: plasma pharmacy, which encompasses plasma activation of liquids; these liquids can be further used in contact with cells and tissue^12^. Despite the significant amount of literature on plasma medicine, the applications of plasma-activated liquid media (PALM) have been less explored^9,13,14^. The complexity of the chemical composition of plasma-generated solutions together with their reactivity and stability not only presents an analytical challenge, but also establishes PALM as a unique and synergistic therapeutic approach^12^. As such, the tunable combination of oxidant species in PALM along with the potential systemic use of these new “drugs” actually accesses specific chemical paths and, as a consequence, certain therapeutic effects that would not otherwise be possible with direct plasma treatments alone^15^. Plasma activation of liquids can be carried out through a remote process in which plasma is ignited close to the surface of the liquid^9^. Most papers on the remote treatment of liquids deal with plasma jet sources^16^. The limits of these sources in this context are mainly the lack of homogeneity of the treatment when volumes higher than a few millilitres are to be treated, and the scant control of the chemical composition of the gas phase^17^. The presence of air surrounding the plasma jet is considered an impurity that not only influences the discharge regime and operation, but also could have a serious detrimental effect on the reproducibility and overall performance of the process^18^. When the gas phase is ignited in open air, its chemical composition cannot be finely controlled; thus, a closed chamber surrounding the plasma and liquid is necessary^19^. Few papers in the literature show the use of DBDs ignited in sealed systems; certain configurations use the liquid as part of the electrical system, with the counter electrode either submerged in the liquid or underneath the liquid separated by a barrier.

Emerging anticancer therapies (i.e., immune therapy) are often characterized by several limitations: pathogenesis complications, drug resistance, cytotoxicity to healthy cells and tissues, side effects, inadequate delivery methods to the tumour site, and high recurrence rates of certain cancer types^20^. For these reasons, scientists are looking for alternative and more effective therapies. The success of any novel cancer treatment depends on its ability to selectively target cancer cells while minimizing cytotoxicity to healthy cells. Indeed, over the past decade, CAP has shown promising results against cancer, and clinical trials are already being organized^21,22^. However, the understanding of the anticancer mechanism of plasma-assisted processes is still very limited, so the basic mechanisms need to be addressed. The advantage in working with plasma, compared to working with conventional therapies, is that plasma-based cancer treatments are less likely to cause drug resistance. A variety of combinations of PALM with other chemical agents have been highlighted in the literature in efforts to gain new insights into the mechanisms of plasma effects against cancer^23^. In 2011^24^, Tanaka *et al*. showed that PALM induced apoptosis in glioblastoma cells via a caspase 3/7 pathway. The application of PALM to cancer cells resulted in the downregulation of AKT, thus impacting the signal transduction pathway associated with survival. No impact was observed on healthy brain astrocytes used as a control, suggesting selectivity of this cancer treatment. Further studies^25^ indicated complete downregulation of survival and proliferation signalling networks (e.g., the PI3K/Akt/mTOR survival pathway, MAPK proliferation pathways, CD44 membrane-bound receptor signalling) for *in vitro* and *in vivo* paclitaxel-/cisplatin-resistant ovarian cancer models, and the antitumour effects were presumably due to reactive oxygen species (ROS), which are considered the main players in the mitigation of survival and proliferation^26,27^. Another study considered the impact of PALM on the morphology and proliferation rate of gastric cancer cells^28^. PALM seems to also affect cell motility and colony formation^29^, while the presence of foetal bovine serum (FBS) in cell culture medium was found to play a protective role in cancer cells^30^, probably due to the scavenging effects of ROS, resulting in lower ROS levels in the culture medium.

The use of a plasma jet working in open air smaller gaps between the plasma source and the media, has been demonstrated to result in a stronger anticancer effect^31^ that differs depending on the cell line used^5^.

Optimizing plasma parameters during direct irradiation of cancer cells with plasmas should make possible the induction of immunogenic cell death (ICD), as well as promote apoptosis and triggering specific, protective immune responses systemically^6,32–34^. Briefly, when cells are stressed or are dying after exposure to various physical or chemical stimuli, they can expose or release some signals that can function as either adjuvant or danger signals for the innate immune system^35–41^. As shown in direct plasma treatment of cancer cells, RONS present in the liquid would be responsible for the induction of ICD in tumour cells, characterized by secretion of damage-associated molecular patterns (DAMPs), which ultimately increases the visibility of the killed cells to the immune system^32,42,43^. After direct CAP exposure, as well as in response to chemotherapeutic agents such as anthracyclines, tumour cells expose complexes with the DAMP calreticulin (CRT) on the extracellular side of their plasma membrane at a pre-apoptotic stage^44^. This feature, which is strictly correlated with immunogenicity, allows the use of dying tumour cells with CRT exposed on the surface as a vaccine therapy against cancer. Another DAMP is the release of ATP from dying cells, which in turn activates macrophages^45^. Miller’s research has demonstrated that plasma-delivered ROS and charged species produced in the plasma increase intracellular ROS and induce the exposure of DAMPs^10^. Due to the complex phenomena present at the interface of the plasma and the biological system, it is important to note that all possible *in vivo* and *in vitro* applications of plasma medicine, including the triggering of ICD, are mediated by the presence of biological liquids (e. g., blood, wound exudate, cell culture media). Despite the expanse of literature on PALM-assisted treatment of cancer cells for testing proliferation, apoptosis, motility and colony formation, only a few studies have investigated the induction of cellular DAMP emission stimulated by PALM. A recent paper showed an increased levels of extracellular-facing CRT after exposure to PALM-treated medium, which was formed by treating Dulbecco’s modified Eagle’s medium (DMEM)/F12 medium with a plasma jet (kINPen MED) fed with Ar, in an *in vivo* model of peritoneal spread of pancreatic cancer^46^. In the approach considered in this study, a DBD is ignited on top of the liquid to promote the diffusion of the active plasma species in the liquid contained in Petri dishes ~57 mm in diameter; due to the containment of the plasma source in the Petri dish, tight control of the active species produced is possible without contamination from external air. With this approach, depending on the gas feed used, the plasma can enrich the liquid with selected RONS derived from the cascade reactions of the plasma species diffused in the liquid phase. The chemistry that occurs in liquid media interfaced with CAP as well as the chemical composition of the gas feed depends on several factors^31,47^: the input power of the plasma; the composition of the substrate solutions; the temporal parameters (e.g., plasma exposure time and lifetime of solution post-preparation); the distance between plasma and liquid surface; the surface area of plasma and the volume of liquid used. Compared to jets, the DBD configuration guarantees homogeneous treatments of larger surface areas (i.e., several centimetres) and greater liquid volumes^48,49^. With this approach, it is possible to properly design plasma treatments and, in principle, to select the RONS to be delivered to the liquid for interfering with cell cycle progression as well as triggering DNA damage, apoptosis, and cell membrane damage^50^.

In this paper, after a preliminary characterization of the antiproliferative activity of PALM, its capability to activate ICD will be investigated in metastatic melanoma (MM) and pancreatic cancer (PDAC) models. Moreover, by using a sealed plasma reactor and by feeding the plasma with O_2_ in principle, it should be possible to enrich the liquid with ROS instead of reactive nitrogen species (RNS) and study the effects of the PALM on tumour cells, which will contribute to new insights into plasma-assisted ICD. The reason why the role of specific reactive species has been addressed in this paper is that, as shown before, the chemical composition of the PALM plays a pivotal role in its effects. H_2_O_2_ has been identified as a critical player in apoptosis via a caspase-independent apoptotic pathway^51^, and nitric oxide (NO) and RNS species are ubiquitous molecules that play a key role in various physiological and pathological processes, including genotoxic mechanisms, antiapoptotic effects, promotion or inhibition of angiogenesis, limitation of host immune response against tumours, and promotion of metastasis. NO is a short-lived molecule that, when in contact with a complex biological milieu such as a cell culture medium, is rapidly metabolized into nitrite and nitrate after its rapid diffusion through liquid media. This effect limits the biological half-life of NO *in vitro* to less than a second, whereas the concentrations of NO relevant for cellular signalling can persist in phosphate-buffered saline for an hour^52^. Thus, the role of NO-related compounds in biochemistry should not be underestimated. Quantification of such components can indirectly give an estimation of the amount of NO produced in a DBD. It has been known for at least a decade that the nitrite anion (NO_2_^−^) can act therapeutically, most likely as an intracellular precursor source of NO^53^. The present paper investigates the effect of H_2_O_2_ and NO_2_^−^ produced in the plasma on the survival of MM and PDAC cells and the role of these molecules in stimulating DAMP secretion from tested tumour cells. By demonstrating the efficacy of PALM in stimulating DAMP emission, the applicability of plasma pharmacy for cancer treatment can be dramatically extended even to internal tumours that are difficult to reach when using topical approaches.

## Materials and Methods

### Experimental set up

**Figure 1:**
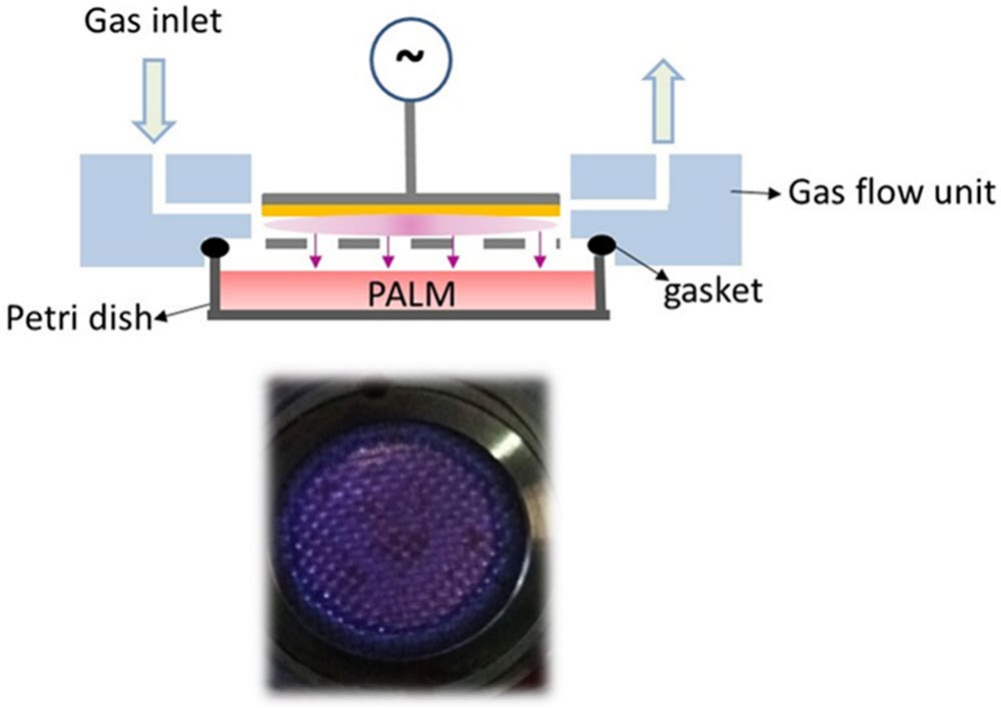
(top) Schematic overview of the experimental set up; (bottom) End-on view of microdischarges produced in air. In yellow, the dielectric.

Figure 1 shows a schematic diagram of the PetriPlas^+^ source utilized in this research. This particular DBD setup was designed at the Leibniz Institute for Plasma Science and Technology (INP) in collaboration with co-authors of this paper (E.S., NANOTEC, Bari, ITA). The source consists of aluminium housing with a ground stainless steel grid electrode. The high voltage electrode, 4 mm from the grid, is made of a copper disc (30 mm dia.) covered with quartz. A picture of the discharge ignited between the copper electrode and the grid is shown at the bottom of Fig. 1.

The PetriPlas^+^ source is designed for operation with commercial Petri dishes 57 mm in diameter. A properly designed plexiglass chamber (flow unit) provides gas connectors and sockets and allows the uniform evacuation of the plasma region and the gap between the source and the liquid. The feed gas is ported in the discharge through an orifice and pumped through a second orifice positioned at the opposite side (longitudinal injection). The discharge unit is placed on the Petri dish as shown in Fig. 1, with the dish fitting exactly the small volume between the discharge and the flow units. A silicone gasket is used to seal the flow unit and the upper rim of the Petri dish. In our experiments, 3 ml of culture medium were  added to the Petri dish with a liquid/gas interface 2 mm from the ground grid. A dry diaphragm pump (Pfeiffer) is used to keep the working pressure constant, measured by an MKS Baratron. Experiments were carried out at 750 mTorr and room temperature, with a flow rate of 0.5 l·min-1 of either synthetic air (Air Liquide, 99.999%) or O_2_ (Air Liquide, 99.999%). The gas flow rates were controlled with MKS mass flow controllers. Before any treatment, the flow unit was purged for 1 min with the feed gas to reduce the presence of impurities in the DBD region. The AC (6 kHz) power supply used in this study was connected to a TG1010A programmable 10 MHz DDS TTi function generator, which allows variation in the sinusoidal voltage, amplitude and frequency of the input power for on/off discharge pulsing. Electric parameters were monitored with a Tektronix TDS 2014 C digital oscilloscope and a Tektronix P6915A HV probe. The transported charges (Q) in the discharge space are determined indirectly through a 100 nF capacitor. When the voltage is high enough to cause a breakdown of the gas gap, the voltage across the measuring capacitor is proportional to the transported charges in the discharge space. Therefore, a charge–voltage (Q-U) plot resembling a closed parallelogram, i.e., a Lissajous figure, is obtained. The area of the parallelogram is equal to the energy deposited in the cycle discharge^54^. The plasma was pulsed with a 50% duty cycle, with a time on (ton) of 50 ms over a period (ton + toff) of 100 ms. The dissipated energy is calculated by multiplying the mean energy per cycle by the ton. The 13 kVpp value of the applied voltage was selected to achieve stable operation with the liquid film and uniformly distribute the microdischarges in the interelectrode gap. This value, along with the frequency (6 kHz) of the applied field, was kept constant in all the experiments. The dose energy was calculated by dividing the dissipated discharge energy by the area of the copper electrode. PALM were produced with either synthetic air or O_2_ at 1 or 3 min of treatment time. Table 1 lists the different experimental conditions investigated with and without the liquid in the Petri dish.

**Table 1:** Experimental parameters.

Sample	Liquid	Gas precursor	Treatemnt time (min)
Air 1 min	no	Synthetic Air	1
Air 1 min liq	yes	Synthetic Air	1
O_2_ 1 min	no	O_2_	1
O_2_ 1 min liq	yes	O_2_	1
O_2_ 3 min	no	O_2_	1
O_2_ 3 min liq	yes	O_2_	1

The voltage (13 KVpp), frequency (6 kHz), pulsing period (100 ms) and ton (50 ms) were kept constant in all conditions.

### H_2_O_2_ detection

A Spectroquant® Hydrogen Peroxide Test was used for the detection of H_2_O_2_ in the cell culture medium. In the presence of a phenanthroline derivative, hydrogen peroxide reduces copper (II) ions to copper (I) ions, which results in the formation of an orange-coloured complex that can be measured photometrically at 445 nm. The samples were analysed within 10 min after plasma processing.

### NO_2_^−^ detection

Nitrites were detected by means of the Griess assay (test kit Spectroquant); nitrite in acid solution reacts with sulphanilic acid to form a diazonium salt, which in turn reacts with N-(1-naphtyl)-ethylenediamine dihydrochloride to form a red-violet azo-dye that can be measured at 525 nm.

### Cell culture

The human PDAC cell line PANC-1 was purchased from ATCC^®^, Hmel1 MM cells (from human sporadic melanoma biopsy specimens, with BRAF mutations) were established as described by Zanna *et al*.^55^, and HBL MM cells (BRAF wild type) were a gift from Prof. G. Ghanem, University of Bruxelles, Belgium. All cell lines were grown in high-glucose DMEM supplemented with 10% (v/v) FBS, 1% (v/v) l-glutamine, and 1% (v/v) penicillin/streptomycin in a humidified atmosphere at 37 °C and containing 5% CO_2_. All materials for cell culture were purchased from EuroClone, Italy.

### Cell imaging

After 5000 cells/well were seeded in 96-well plates and reached 80% confluence, they were incubated with PALM (in O_2_ 1 min, O_2_ 3 min or air 1 min conditions) and viewed on an inverted light microscope at 48 h after treatment. Images were acquired with an inverted microscope Olympus CKX41 (10 X magnification).

### Cell proliferation assay

A total of 5000 cells/well were seeded in 96-well plates and cultured to 80% confluence. The antiproliferative effect of PALM (in O_2_ 1 min, O_2_ 3 min or air 1 min conditions) on PANC-1, Hmel1 and HBL cells was evaluated with the 3-[4,5-dimethylthiazol-2-yl]-2,5-diphenyltetrazoliumbromide (MTT) assay at 48 h after treatment. The results are shown as cell viability (%) histograms of the mean of four different experiments.

### Determination of ROS generation by H_2_DCFDA staining

After cells (80% confluence) were treated with PALM (O_2_ 3 min) for 3 h, they were washed with PBS and incubated for 30 min at 37 °C in the dark with 10 μM 2′,7′-dichlorodihydrofluorescein diacetate (H_2_DCFDA) to assess ROS levels in cells. Upon cleavage of the acetate groups by intracellular esterases and oxidation by ROS, the non-fluorescent H_2_DCFDA is converted to the highly fluorescent 2′,7′-dichlorofluorescein (DCF). The cells were analysed for fluorescence intensity by flow cytometry (FCM). Untreated cells not loaded with the dye were used as a negative control to examine cellular autofluorescence. CM-H_2_DCFDA was provided by Molecular Probes (Life Technologies, USA). Data are reported as histograms of the ratio of ROS measured after PALM treatment (ROS_afterPALM_) to the basal level of ROS (ROS_basal_).

### Apoptosis assay

After PANC-1, Hmel1 and HBL cells at 80% cell confluence were treated with PALM (O_2_ 3 min) for 24 and 48 h, the induction of apoptosis was determined using a FITC Annexin V Apoptosis Detection Kit II (BD Pharmingen, USA) according to the instructions provided by the manufacturer.

### Cellular effector analysis

After human pancreatic and melanoma cancer cells (at 80% cell confluence) were treated with PALM (O_2_ 3 min) for 4, 24, 48 and 72 h, the protein level of CRT was analysed by FCM. After treatment, cells were harvested and washed in ice-cold PBS (pH 7.4), resuspended in 100 µL of PBS with 3% bovine serum albumin (BSA). CRT membrane expression was evaluated by incubating both treated and untreated cells with polyclonal anti-CRT antibody (Thermo Fischer Scientific, USA, 1:100 dilution) in 3% BSA in PBS for 30 min at 4 °C. The suspension was centrifuged in 1 mL of 3% BSA in PBS, the pellet was resuspended in 100 µL of 3% BSA in PBS and incubated with FITC goat anti-rabbit IgG secondary antibody (BD Pharmingen™; 1:100 dilution) for 30 min at 4 °C. After two wash steps, cells were resuspended in 200 µL of 3% BSA in PBS and assessed for CRT protein expression using a FACScan flow cytometer. The data analysis was carried out with CellQuest software (Becton Dickinson, NJ). The LC3-I and II protein levels in human pancreatic and melanoma cancer cells treated for 72 h with activated DMEM under PALM conditions (O_2_ 3 min) and in untreated control cells were analysed as previously described^56^ by western blot analysis. In particular, the protein extracts were obtained by homogenization in RIPA buffer and then treated with 1 mM phenylmethylsulphonyl fluoride (PMSF). Blot detection and image analysis were performed with ChemiDoc™ Imaging Systems and ImageLab software (Bio-Rad-USA), respectively. The monoclonal antibody anti-LC3 A/B I and II was provided by Cell Signaling Technology. The secondary antibody used was an HRP-conjugated rabbit antibody (Bio-Rad Laboratories, USA). All experiments were performed in triplicate.

### Determination of ATP release from cells

After cells reached 80% confluence, the levels of ATP release from untreated cells and cells incubated with PALM for 4, 24, 48 and 72 h were evaluated using an ATP Determination Kit, 200-1,000 assays (Thermo Fischer Scientific-USA) according to the instructions provided by the manufacturer.

### Statistical analysis

Statistical significance was calculated using a two-way analysis of variance (ANOVA) followed by Bonferroni post hoc tests (GraphPad Prism ver. 5). Data are indicated as follows: *p < 0.05, **p < 0.01, and ***p < 0.001.

## Results

### Electrical characterization of plasma

**Figure 2:**
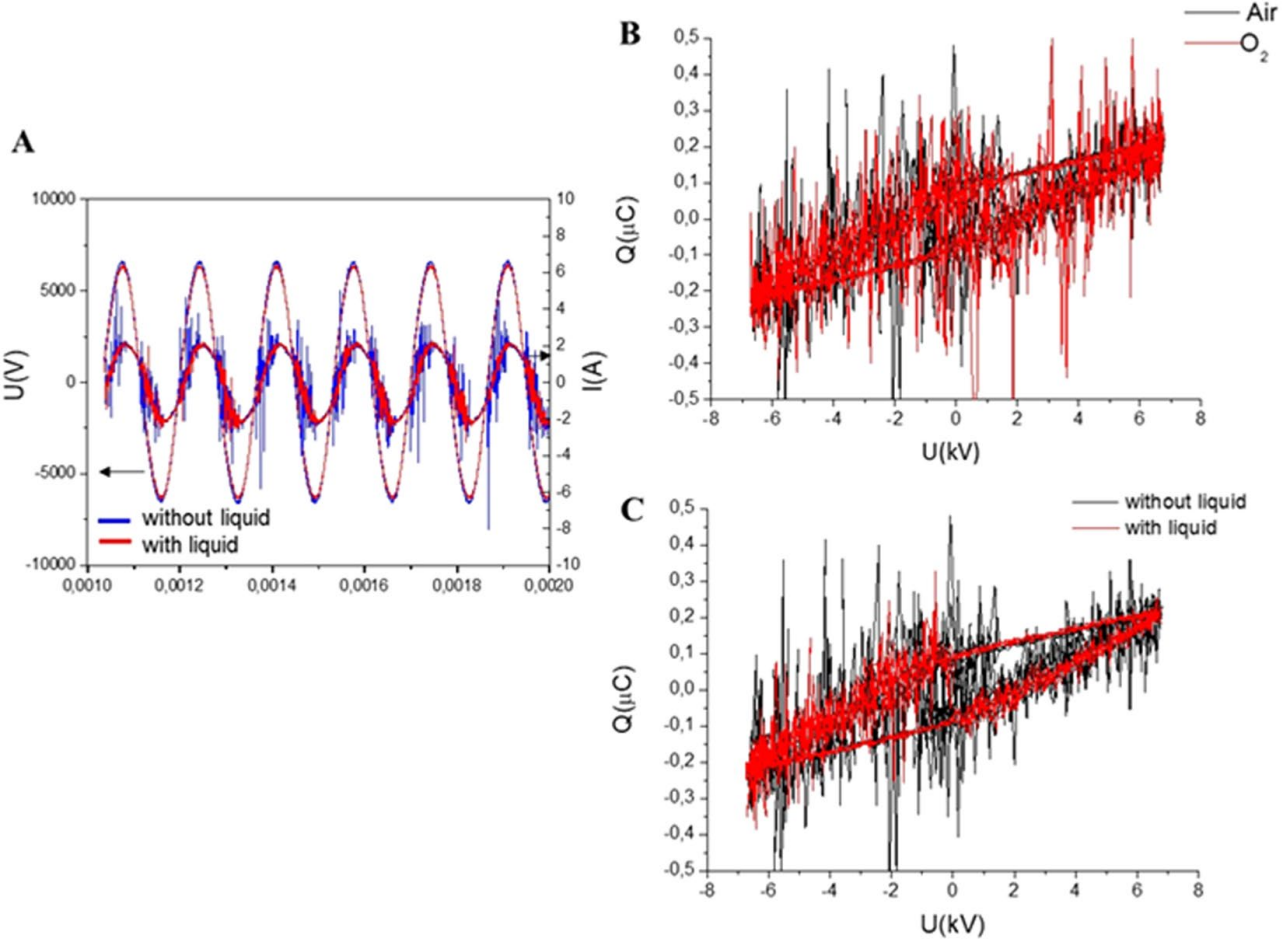
(**A**) Current and voltage signals and Lissajous figures showing the discharge power in air and O_2_ without liquid (**B**) and of the DBD operating with and without liquid with air as the carrier gas (**C**).

Typical waveforms of discharge voltage and discharge current for air and O_2_ carrier gases for discharge operating with and without liquid are shown in Fig. 2. Microdischarge activity is indicated in Fig. 2A by short peaks on top of the capacitive current, I(A), and observed only when the voltage in the gap reaches the average discharge voltage. Due to the high amplitude of the applied voltage, the formation of microdischarges is favoured^57^. The plasma source was 2 mm from the surface of the liquid, and the bottom of Fig. 1 shows the discharge switched on in synthetic air. Along with the current–voltage waveforms, Fig. 2B,C also shows Lissajous figures (Q–U plots). These plots were used to determine the energy dissipated into the plasma after one cycle of high voltage. The area of the Lissajous figure represents the energy of the electrical discharge in one AC cycle.

Under the investigated experimental conditions, the energy dissipated in the discharge was the same with and without the liquid underneath, as summarized in Table 2. In all conditions, only the treatment time influenced the energy dissipated in the system.

**Table 2:** Electrical parameters of the applied discharges.

Sample	E cycle (mJ)	Ediss (J)	Energy dose (J/cm^2^)
Air 1 min	0.99 ± 0.04	177 ± 7	25.0 ± 1.0
Air 1 min liq	1.05 ± 0.07	188 ± 13	27 ± 2
O_2_ 1 min	1.01 ± 0.01	181 ± 2	25.6 ± 0.3
O_2_ 1 min liq	1.07 ± 0.05	191 ± 9	27.0 ± 1.3
O_2_ 3 min	1.01 ± 0.01	543 ± 6	76.9 ± 0.8
O_2_ 3 min liq	1.07 ± 0.05	573 ± 30	81 ± 4

### Chemical analysis of PALM

**Figure 3:**
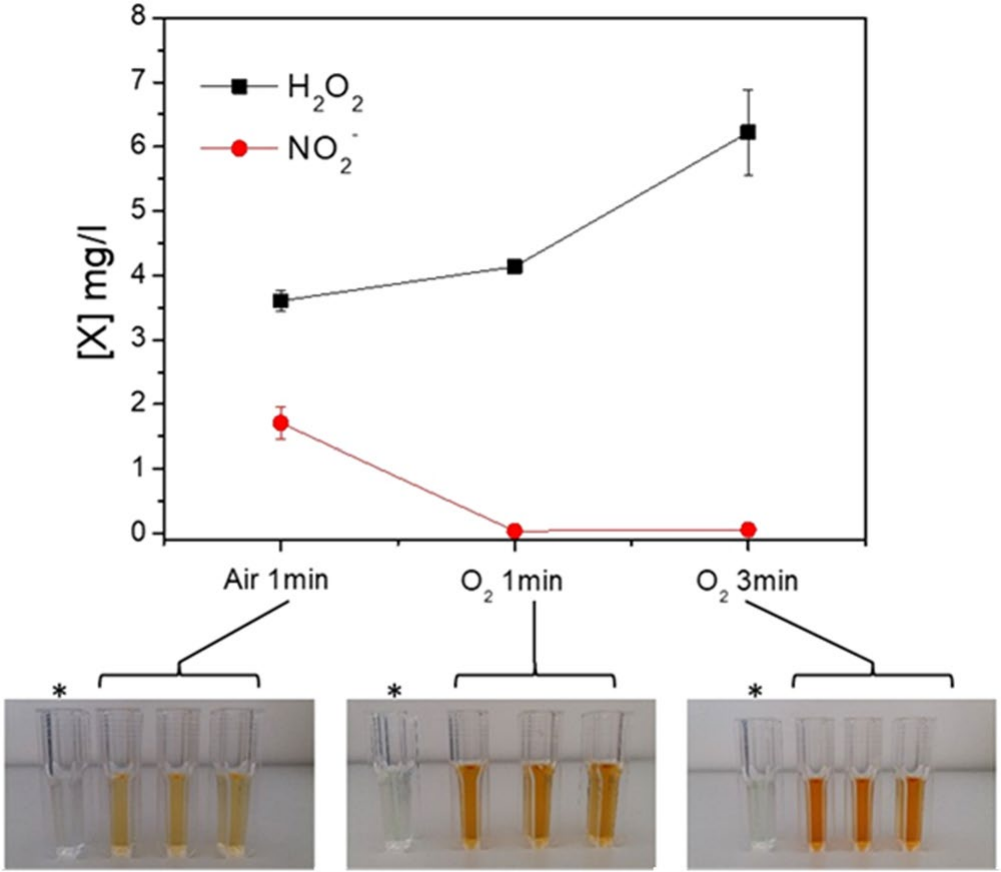
Concentration of hydrogen peroxide and nitrites revealed in the PALM in three different experimental conditions: Air 1 min (0.5 slm Air, 13 kV, 1 min, 100 msT, 50% DC); O_2_ 1 min (0.5 slm O_2_, 13 kV, 1 min, 100 ms of period T, 50% DC); and O_2_ 3 min (0.5 slm O_2_, 13 kV, 3 min, 100 msT, 50% DC). Photos: pictures corresponding to a PALM solution obtained from the three different plasma conditions and treated with the H_2_O_2_ kit. *Control: untreated cell culture treated with the H_2_O_2_ kit.

The chemical composition of the gas feed and the energy dose are expected to influence the chemical composition of the PALM. The graph reported in Fig. 3 shows the concentration of H_2_O_2_ and NO_2_^−^ produced in three different PALM. It is evident that the H_2_O_2_ concentration increases with the amount of O_2_ in the feed as well as with the energy density (i.e., increase in the colour intensity in the solutions contained in the cuvettes with respect to the control; bottom of Fig. 3); it is also evident that the concentration of NO_2_^−^ remains below the detection limit in the PALM unless N_2_ (component of air) is fed into the system.

### Cell viability and types of cell death after incubation with PALM

**Figure 4:**
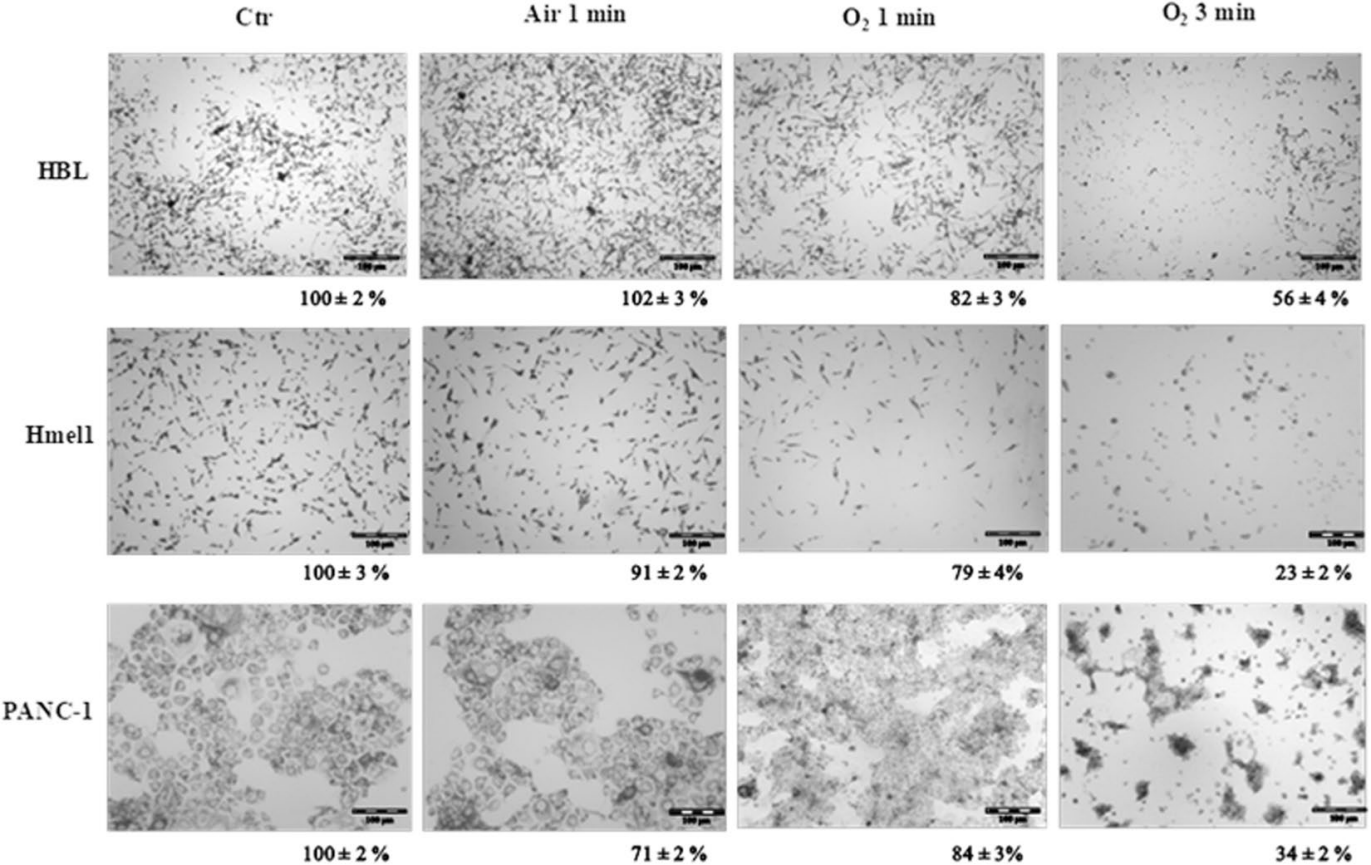
Images of HBL, Hmel1 and PANC-1 cells treated for 48 h with PALM produced in O_2_ 1 min, O_2_ 3 min and air 1 min experimental conditions. The cell viabilities (%) indicated by the MTT assay are reported. Images were acquired on an Olympus CKX41 inverted microscope.

The MM cell lines HBL and Hmel1 and the PDAC cell line PANC-1 were treated with three different PALM (O_2_ 1 min, O_2_ 3 min or air 1 min) and then observed under a microscope to evaluate any morphological changes. After 48 h of incubation, a strong reduction in viable cells treated with PALM O_2_ 3 min was observed compared to the number of viable cells in the untreated group (Fig. 4). This was most evident in Hmel1 cells compared to HBL and PANC-1 cells. The other treatment conditions (O_2_ 1 min and air 1 min) had a lower impact on morphology and viability in all three cell models. For Hmel1 cells, exposing these cells to PALM O_2_ 1 min resulted in a slightly lower number of adherent cells with respect to the control cells (untreated cell culture medium) and cells treated with PALM air 1 min. This lead to the hypothesis that ROS exert a greater effect than RNS, and, in order to harness this activity and improve the efficacy of PALM, we decided to conduct all the further *in vitro* experiments with PALM O_2_ 3 min.

**Figure 5:**
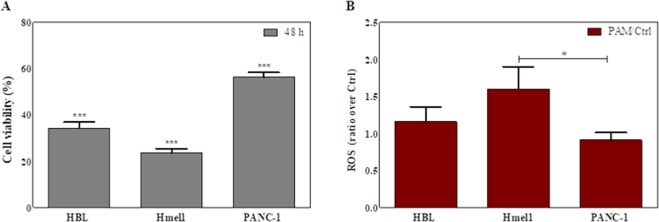
(**A**) Cell viability of HBL, Hmel1 and PANC-1 cells after 48 h of incubation with PALM O_2_ 3 min; (**B**) ROS_afterPALM_/ROS_basal_ ratio in HBL, Hmel1 and PANC-1 cells after treatment with PALM O_2_ 3 min for 3 h. Data from 3 sample replicates are shown (mean ± SD, ***p < 0.001, *p < 0.05).

The MTT assay results reported in the histogram in Fig. 5A as % cell viability compared to control cells confirmed a stronger reduction in cell viability in MM cells than in PDAC cells after a 48-h incubation with PALM O_2_ 3 min. In all samples, the reduction in cell viability was statistically significant (p < 0.001). These results seem to be correlated with the ROS levels found in the cell lines under investigation before and after treatment with PALM (Fig. 5B). PANC-1 cells showed a higher basal level of ROS than Hmel1 and HBL cells (data not shown). When PALM treatment increased intracellular ROS production compared to that in control cells, the result was a greater cytotoxic effect. In fact, the calculated ratio ROS_afterPALM_/ROS_basal_ reported in Fig. 5B is greater in Hmel1 cells than in HBL and PANC-1 cells. The difference in the ratio between Hmel1 and PANC-1 cells was significantly different (p < 0.05).

**Figure 6:**
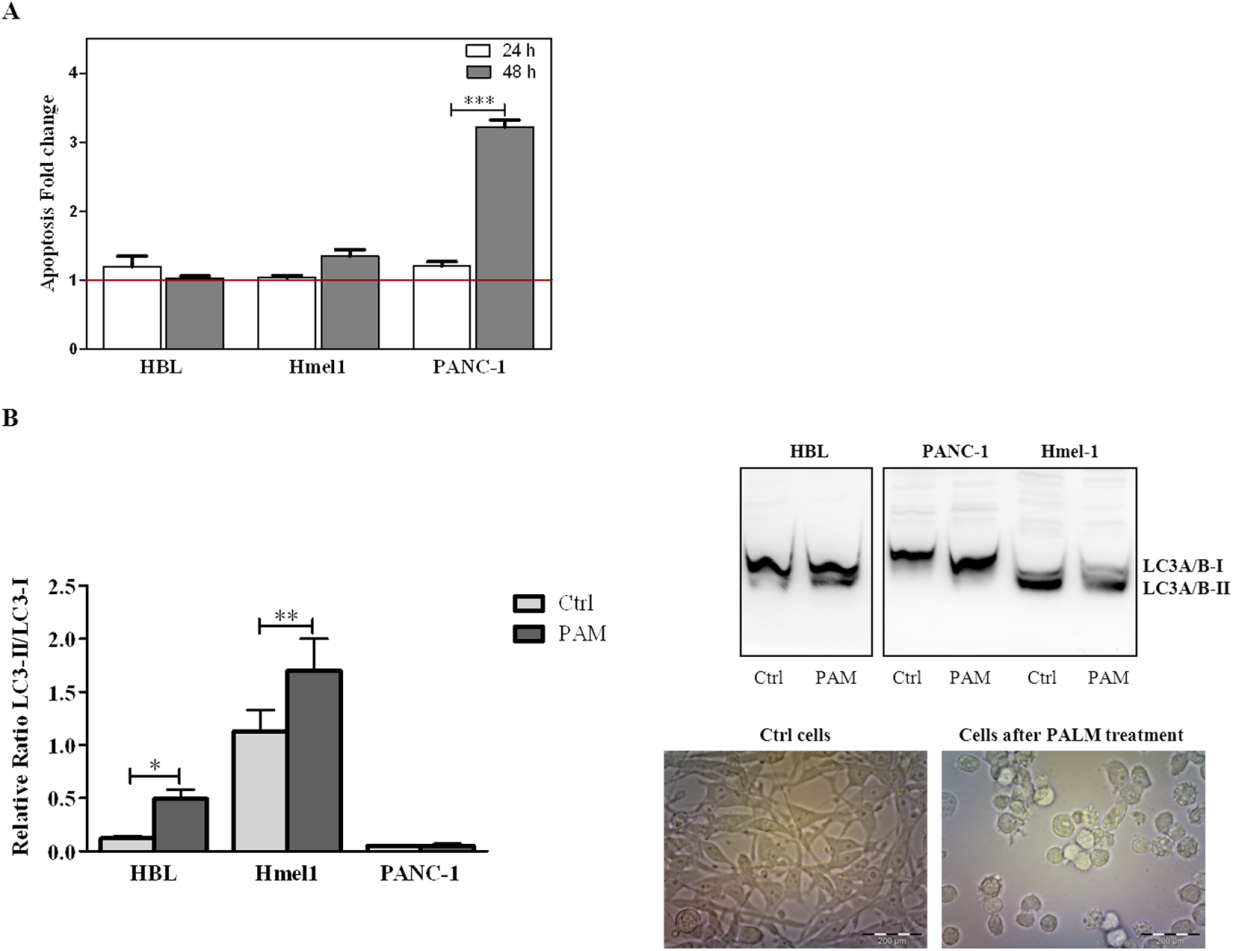
(**A**) Apoptosis of HBL, Hmel1 and PANC-1 cells after 24 h or 48 h of PALM treatment was detected by the Annexin V/PI assay and is expressed as fold change compared to control cells. (**B**) Autophagy was determined by western blotting and expressed in the histogram as the LC3-II/LC3-I ratio. Protein extracts of HBL, Hmel1 and PANC-1 cells were obtained at 72 h after treatment with PALM. The protein bands are reported, and the stain-free method was used to normalize the values. Data from 3 sample replicates are shown (mean ± SD, ***p < 0.001, **p < 0.01, *p < 0.05). The full-length blots are presented in Supplementary Fig. 1. The samples were obtained from the same experiment, and the blots were processed in parallel. Images of Hmel1 cells before and after PALM treatment are shown.

To characterize the types of cell death responsible for the anticancer activity of PALM, all cell lines were treated with PALM for 24 h and 48 h and then stained with Annexin V/PI. Flow cyotmetry analysis showed that PANC-1 and Hmel1 cells exposed to PALM for 48 h died because of apoptosis. This phenomenon was markedly evident in the PDAC model, while HBL cells did not undergo this mechanism of cell death. The results are reported in the histogram in Fig. 6A as fold change of apoptosis (early and late) in the treated group *vs* control group for every cell line after 24 h and 48 h of treatment with PALM.

We also evaluated whether autophagy was involved in the observed cell death. Protein extracts from cells treated with PALM for 72 h were screened for LC3 by western blotting. The detection of LC3 conversion from LC3-I to LC3-II, when correlated with the number of autophagosomes, is an indication of autophagy^58^. The results, shown in Fig. 6B as the ratio of LC3-II *vs* LC3-I, demonstrated that the activation of autophagy represented by the increase in the LC3-II/LC3-I ratio in the histogram and by the strong LC3-II band in the western blot occurred only in HBL and Hmel1 cells incubated with PALM for 72 h; in particular, LC3-II was 4 and 1.3 times higher than LC3-I in HBL and Hmel1 cells, respectively, after PALM treatment. The optical images of Hmel1 cells in Fig. 6B show autophagic cells only after PALM treatment. In contrast, in PANC-1 cells, autophagy was not observed in either control cells or cells treated with PALM.

These data show that PALM induced a reduction in cell viability through two different types of cell death, apoptosis and autophagy, which could occur simultaneously.

### Activation of immunological cell death (ICD) after PALM treatment

**Figure 7:**
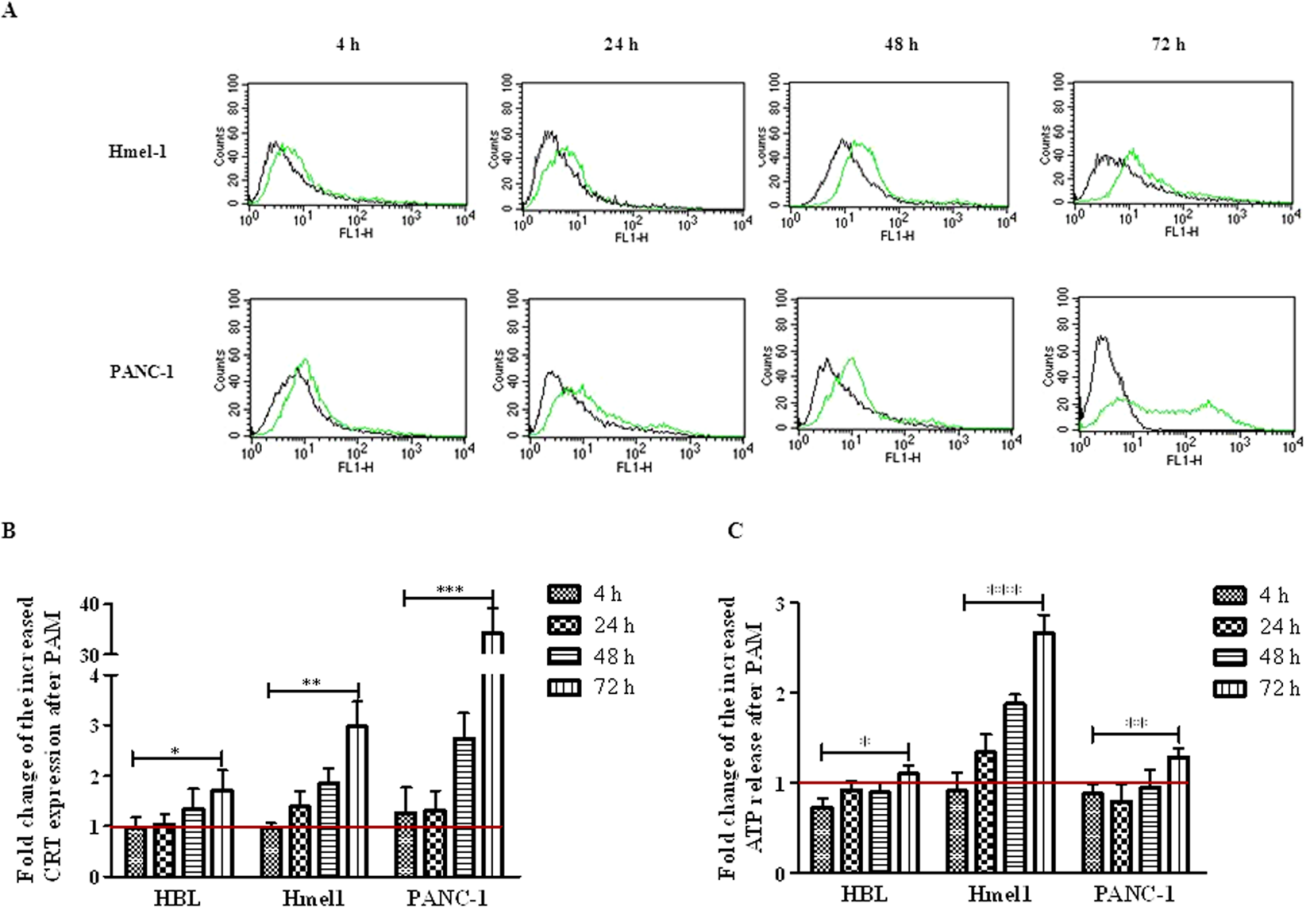
(**A**) CRT expression in Hmel1 and PANC-1 cells after incubation with PALM for 4, 24, 48 and 72 h was determined by CFM. (**B**) Fold change of CRT expression in HBL, Hmel1 and PANC-1 cells treated with PALM compared to that in untreated cells; (**C**) Fold change of ATP released by HBL, Hmel1 and PANC-1 cells after 4, 24, 48 and 72 h with PALM treatment compared to that from untreated cells. Data from 3 sample replicates are shown (mean ± SD, ***p < 0.001, **p < 0.01, *p < 0.05).

To investigate the activation of ICD in all tumour cell lines treated by PALM, the exposure of the chaperone CRT to the extracellular environment and the release of ATP from dying cells were evaluated. Hmel1 and PANC-1 cells showed much more CRT exposure than HBL cells (Fig. 7A,B). A comment is necessary for PANC-1 cells: after 72 h, even though a strong increase in CRT was shown by FCM (Fig. 7B), the histogram in Fig. 7A indicated that a portion of the cell population seemed to have less CRT, maybe for the presence of a resistance mechanism. To investigate this hypothesis, the release of ATP was evaluated, and it was revealed that in Hmel1 cells, its release into the medium markedly increased as a function of time (Fig. 7C), while in PANC-1 cells, there was release only at 72 h after PALM treatment. HBL cells seemed to be the most resistant to the activation of ICD; in fact, neither the displacement of CRT on the membrane nor the release of ATP were evident at short time points after treatment, and only a 72 h of incubation with PALM did these cells show slightly increases of CRT on the membrane and the beginnings of ATP release.

## Discussion

It is known that MM and PDAC are the cancer pathologies with the worst prognosis. The low survival of patients affected by these two diseases depends on several factors, such as diagnosis of cancer at advanced stages, presentation of metastatic lesions, inability to treat with surgery, and the low response to chemotherapeutic or radiotherapy treatments. In particular, the establishment of either innate or acquired resistance, which are due to a tumour microenvironment that reduces the penetration of drugs or ionizing radiations (i.e., the dense desmoplastic stroma of PDAC)^59^, is among the most accredited causes of patients exhibiting no response to conventional treatments such as chemotherapy, radiotherapy and new biological approaches^60,61^. When exploring new therapeutic strategies, particular attention should be paid to possible indirect activities such as the induction of ICD, along with the study of direct anticancer activity. When cancer cells are dying, they can induce the production and release of DAMPs that are responsible for the increase in visibility of the tumour by the immune system’s cells that, in turn, initiate a response against the tumour.

The hypothesis of this work is to use a physical tool, cold plasma, to activate a medium (e.g., PALM) that might impact cell proliferation, as other authors have already demonstrated^27,62^, by inducing cell death via ICD. In the two cancer models, PDAC and MM, after preliminarily establishing the optimal conditions for preparing PALM, we characterized the antiproliferative effectiveness of PALM together with the mechanisms of cell death induced and the possibility of activating ICD.

In particular, we observed that when air was used as feed, almost no effect occurred on cell viability, most likely due to the minimal presence of H_2_O_2_ found in the cell culture medium and the high concentration of nitrites, the latter of which were completely absent in PALM prepared with O_2_ as the gas feed. Nitrites, that can almost be considered products of the reaction of NO with other components present in the PALM, were not as effective as H_2_O_2_ against the cancer lines investigated. Among the three different conditions investigated, the best was “O_2_ 3 min”, which was used in all subsequent experiments. Discharges operated by increasing the treatment time up to 3 min dissipated more energy; therefore, an increase in H_2_O_2_ was detected. The formation of long-living species such as H_2_O_2_ can be explained by the short diffusion length of OH radicals in the liquid that leads to recombination reactions. As shown in the literature, more than 60% of the liquid phase H_2_O_2_ was estimated to be generated without a direct plasma effect in the experiment under observation^63^. The OH radicals produced during the plasma process due to the presence of water vapour in the gas phase diffuse through the liquid, producing hydrogen peroxide with different chemical patterns, as shown below:

1 $$\cdot {\rm{OH}}+\cdot \,{\rm{OH}}\to {{\rm{H}}}_{{\rm{2}}}{{\rm{O}}}_{2}\,{\rm{Hydroxyl}}\,{\rm{Radical}}\,{\rm{Recombination}}$$

It is known from radiation chemistry that in the presence of high levels of molecular oxygen, H_2_O_2_ could also be formed by the recombination of hydroperoxyl radicals as follows:

2 $$({\rm{a}})\,{\rm{H}}+{{\rm{O}}}_{{\rm{2}}}\to {{\rm{HO}}}_{{\rm{2}}}\cdot ({\rm{b}})\,{{\rm{2HO}}}_{{\rm{2}}}\,\cdot \to {{\rm{H}}}_{{\rm{2}}}{{\rm{O}}}_{{\rm{2}}}+{{\rm{O}}}_{2}\,{\rm{Hydroperoxyl}}\,{\rm{Radical}}\,{\rm{Recombination}}$$

Hydrogen peroxide was detected in all the experimental conditions investigated in the presence of either air or O_2_. If we focus our attention only on H_2_O_2_, its  concentration inside PALM obtained with discharges carried out for 1 min, regardless of the gas feed used, was the same. However, the results obtained from the cell proliferation experiments demonstrate that, despite containing the same amount of H_2_O_2_ as PALM obtained with O_2_ 1 min, PALM obtained with air 1 min does not affect cancer cell proliferation. These results allow us to assess that active N-containing species, including nitrites, are produced in PALM obtained with air, and these species, in turn, can probably promote a protective effect towards cancer cells. In fact, as reported in the literature, in addition to H_2_O_2_, nitrites are also a potential therapeutic effect  against cancer. Aqueous solutions of sodium nitrite are delivered intravenously or even orally as a therapy^64^. As is well documented in the pertinent literature, nitrites can be reduced to nitric oxide in acidic environment containing low concentrations of oxygen. In cell culture medium, an acidic environment does not develop due to the presence of a buffer that keeps the pH constant in the range 7.4–7.8 regardless of the plasma treatment implemented. Due to the organic components present in cell culture medium, and the presence of cells, when the liquids are kept in contact with the cells, the reactions in the liquid phase involving nitrites can be much more complex than those observed *in vivo*^65^.

In both the MM and PDAC models, the investigation of the antiproliferative activity of PALM (O_2_ 3 min) and the determination of the intracellular accumulation of ROS after 3 h of exposure to PALM showed a marked inhibition of cell proliferation, which was inversely correlated with the intracellular ROS concentration. Although we are aware that our model is only exploratory, based on the 3 cell lines tested, the data seem to indicate that the pancreatic cancer cell line PANC-1, which had the highest basal intracellular level of ROS and the lowest accumulation after treatment, was also less responsive to PALM treatment; however, in the MM cell lines, lower basal ROS values and a higher accumulation after PALM treatment correlated with a stronger response. These data are in agreement with the data in the literature, which showed that ROS cause a reduction in cell proliferation^66–68^.

Upon analysis of the cell death mechanism responsible for the anticancer activity of PALM, we found that PANC-1 cells died during apoptosis, while MM cell lines mainly underwent autophagy. These data are in agreement with those previously reported, underscoring the fact that the MM cell lines are intrinsically autophagic and that this autophagy is higher in the presence of BRAF mutations, as evident in Hmel1 cells^56^. However, it is known that ROS can kill cells through either apoptosis or autophagy^69–71^, but, it was demonstrated for the first time that PALM might inhibit proliferation through various cell death mechanisms.

Furthermore, we investigated whether this new therapeutic approach may also induce ICD activation because the two tumour models chosen are profoundly different in their response to immunotherapy.

Immune checkpoint inhibitor treatment is the definitive gold standard for MM patients carrying the BRAF V600 mutation who no longer respond to BRAF or MEK inhibitors, or have wild-type BRAF. In PDAC, however, this therapeutic approach was not successful, especially when immune checkpoint inhibitors are used as a monotherapy, except in the case of a rare subset of tumours harbouring microsatellite instability (<2%). The poor immunogenicity of PDAC is due to the abundant stroma; this makes this tumour difficult to treat, and reduce the ability to recruit immunosuppressive cells through cancer-associated-fibroblast activation and transforming growth factor β secretion^72,73^.

Consequently, our study, which was focused on ICD, reveals that PALM can cause a type of cell death that induces activation of the innate immune system through the release of DAMPs, and thus can ultimately provoke an all-encompassing antitumour immune response in tumour pathologies that are less responsive to classical immunotherapy^43,74–76^.

We demonstrated that both MM and PDAC tumour models, when exposed to PALM, showed an increase in CRT exposure on the plasma membrane and elevated ATP release, two crucial DAMPs for ICD; the intensities of the cells’ responses were different. As far as the possible mechanisms responsible for the induction of ICD, as it relates to the well-known link between oxidative stress through increased intracellular ROS levels and the induction of ICD^10,77^, autophagy and apoptosis could also be involved. In our experimental conditions, cells were incubated with PALM at 1 h after its production, and in consideration of future clinical applications, it is not possible to prepare PALM and immediately administer it to the patient. Thus, even if we agree with other authors that ROS are involved in ICD, our suggestion is that its induction depends on long-lived species (H_2_O_2_, NO, etc.) rather than short-lived reactive species (∙OH, etc). Autophagy promotes ICD and antitumour immune responses through the regulation of ATP release, while it has no impact on the surface exposure of CRT^78–80^. In fact, in Hmel1 cells, which showed the strongest PALM-induced autophagy response, a higher levels of ATP release were found. Regarding CRT, we found that the highest amount of membrane exposure was in the PDAC model, in which cells died due to apoptosis induction. This evidence agrees with reports in the literature that state that the cytosolic concentration of CRT increases under apoptotic stress conditions^81,82^. Furthermore, despite the differences in the PALM preparation protocols, our results are in agreement with those reported by Liedtke *et al*., who demonstrated that plasma jet-treated medium increased the exposure of CRT on tumour cells in a murine model of peritoneal spread of pancreatic cancer^46^.

Thus, our data suggest that, in MM and PDAC, treatment with PALM activates ICD; therefore, if this system is properly validated in larger cell line panels and *in vivo* models, PALM produced with O_2_, could serve as an ICD inducer that can activate the innate immune system to target these tumours.

In fact, it is known that DAMPs, including toll-like receptors (TLRs), can be recognized by receptors on macrophages and dendritic cells, and initiate a broad immune response through a well-defined mechanism, resulting in the increased presentation of cell-associated antigens to CD4+ and CD8+ T lymphocytes^43^.

Moreover, to harness the immune system to preserve survival and increase the efficacy of immunotherapy in low immunogenic cancers, the combination of PALM, as a potential ICD inducer, and immunotherapy (e.g., anti-CD47 antibody and anti-CTLA-4) may be suitable to improve therapeutic outcomes, as suggested by the literature^83–86^. Indeed, this combination might result in a bidirectional synergy between classical immunotherapy, which is efficient for patients with “T cell-hot” tumours, and PALM-dependent induction of ICD, which could facilitate an initial T cell-driven anticancer immune response potentially sustained by the presence of immune checkpoint blockers^85,87^.

## Conclusion

Most plasma medicine papers dealing with cancer treatments are mainly focused on the duration of the direct plasma treatment rather than on other plasma parameters as a means of showing how to improve the efficacy of the treatment. To the best of our knowledge, our paper is the first describing the indirect treatment approach mediated by PALM treated with different gas feeds in a closed DBD system to distinguish the effects of ROS from those of RNS on tumour cells. We used only O_2_ in the feed and a sealed plasma chamber to avoid contamination of external air and limit the formation of RNS in PALM. Moreover, for prospective work, our study should strengthen the knowledge base on the activity of ICD inducers and should serve as a platform for the identification of reliable biomarkers for monitoring the induction of ICD by PALM, which could be used in the selection of patients for appropriate treatments.

## Supplementary information

Supplementary Figure 1
