# Supplementary material for: Plasma-activated medium triggers cell death and the presentation of immune activating danger signals in melanoma and pancreatic cancer cells
Source: Sci Rep. 2019 Mar 11;9:4099. doi: 10.1038/s41598-019-40637-z (PMC6411873; doi:10.1038/s41598-019-40637-z)
Supplement: Supplementary file 1 — Supplementary Figure 1 [file 41598_2019_40637_MOESM1_ESM.docx]

**Plasma-activated medium triggers cell death and the presentation of immune activating danger signals in melanoma and pancreatic cancer cells**

Amalia Azzariti^1,*^ Rosa Maria Iacobazzi^1^, Roberta Di Fonte^1^, Letizia Porcelli^1^, Roberto Gristina^2^, Pietro Favia^2,3^, Francesco Fracassi^2,4^, Ilaria Trizio^4^, Nicola Silvestris^5^, Gabriella Guida^6^, Stefania Tommasi^7^, Eloisa Sardella^2,*^

^1^Experimental Pharmacology Laboratory, IRCCS Istituto Tumori Giovanni Paolo II, Viale O. Flacco, 65, 70124 Bari, Italy;

^2^Institute of Nanotechnology, National Research Council of Italy (CNR-NANOTEC), c/o Department of Chemistry, University of Bari “Aldo Moro” Via Orabona 4, Bari 70126, Italy;

^3^Department of Biosciences, Biotechnologies and Biopharmaceutics, University of Bari “Aldo Moro” Via Orabona 4, Bari 70126, Italy;

^4^ Department of Chemistry, University of Bari “Aldo Moro” Via Orabona 4, Bari 70126, Italy.

^5^Scientific Direction, IRCCS Istituto Tumori Giovanni Paolo II, Viale O. Flacco, 65, 70124 Bari, Italy;

^6^Department of Basic Medical Sciences, Neurosciences and Sense Organs - University of Bari “Aldo Moro” Via Orabona 4, Bari 70126, Italy;

^7^Molecular Diagnostics and Pharmacogenetics Unit - IRCCS Istituto Tumori Giovanni Paolo II, Viale O. Flacco, 65, 70124 Bari, Italy.

* Corresponding authors:

AmaliaAzzariti

Experimental Pharmacology Laboratory

IRCCS Istituto Tumori Giovanni Paolo II

Viale O. Flacco, 65

70124 Bari, Italy

Tel: +39-080-5555986

Email: a.azzariti@oncologico.bari.it

Eloisa Sardella

CNR, Institute of Nanotechnology (CNR-NANOTEC)

c/o Dept. Chemistry, University of Bari

via Orabona, 4

70126 Bari, Italy

Tel.: +39-080-5442295

Fax.: +39-0805443405

Email: eloisa.sardella@cnr.it

**Supplementary Figure 1.** (A) Full-length blot of HBL (see bands in dashed rectangle); (B) Full-length blot of PANC-1 and Hmel1 (see bands in dashed rectangle).
